# Convenient Auto-Processing Vector Based on Bamboo Mosaic Virus for Presentation of Antigens Through Enzymatic Coupling

**DOI:** 10.3389/fimmu.2021.739837

**Published:** 2021-10-14

**Authors:** Ming-Hao Yang, Chung-Chi Hu, Chi-Hzeng Wong, Jian-Jong Liang, Hui-Ying Ko, Meng-Hsun He, Yi-Ling Lin, Na-Sheng Lin, Yau-Heiu Hsu

**Affiliations:** ^1^ Graduate Institute of Biotechnology, National Chung Hsing University, Taichung, Taiwan; ^2^ Advanced Plant Biotechnology Center, National Chung Hsing University, Taichung, Taiwan; ^3^ Institute of Biomedical Sciences, Academia Sinica, Taipei, Taiwan; ^4^ Institute of Plant and Microbial Biology, Academia Sinica, Taipei, Taiwan; ^5^ Biomedical Translation Research Center, Academia Sinica, Taipei, Taiwan

**Keywords:** *Bamboo mosaic virus* (BaMV), chimeric virus particle (CVP), nanoparticle, virus-like particle (VLP), antigen presentation platform, *Tobacco etch virus* protease (TEVp), sortase A, *Japanese encephalitis virus* (JEV)

## Abstract

We have developed a new binary epitope-presenting CVP platform based on bamboo mosaic virus (BaMV) by using the sortase A (SrtA)-mediated ligation technology. The reconstructed BaMV genome harbors two modifications: 1) a coat protein (CP) with N-terminal extension of the tobacco etch virus (TEV) protease recognition site plus 4 extra glycine (G) residues as the SrtA acceptor; and 2) a TEV protease coding region replacing that of the triple-gene-block proteins. Inoculation of such construct, pKB5G, on *Nicotiana benthamiana* resulted in the efficient production of filamentous CVPs ready for SrtA-mediated ligation with desired proteins. The second part of the binary platform includes an expression vector for the bacterial production of donor proteins. We demonstrated the applicability of the platform by using the recombinant envelope protein domain III (rEDIII) of Japanese encephalitis virus (JEV) as the antigen. Up to 40% of the BaMV CP subunits in each CVP were loaded with rEDIII proteins in 1 min. The rEDIII-presenting BaMV CVPs (BJLPET5G) could be purified using affinity chromatography. Immunization assays confirmed that BJLPET5G could induce the production of neutralizing antibodies against JEV infections. The binary platform could be adapted as a useful alternative for the development and mass production of vaccine candidates.

## Introduction

Virus-like particles (VLPs) or chimeric virus particles (CVPs) have been utilized extensively as effective scaffolds for the presentation of epitopes or antigens in the development of vaccine candidates (1–4). The antigens displayed on the surfaces of VLPs and CVPs can stimulate strong and long-lasting immune responses, since the VLPs and CVPs are assembled from hundreds or thousands of highly ordered coat proteins (CPs) which present the antigens repeatedly for the immune system. Furthermore, the size, shape, and rigidity of most viruses and the derived VLPs and CVPs are suitable to enter the lymphatic system for their uptake by antigen presenting cells of the immune system (5). Together with the relative ease in manipulating the surface properties (6, 7), these features make VLPs and CVPs ideal alternative platforms for vaccine development.

VLPs and CVPs derived from plant viruses exhibit additional advantages as scaffolds for antigen presentation, compared to systems based on non-plant viruses. Plant viruses are not pathogens to animals and humans, thus pose less biosafety threats. In addition, most plant viruses encode only a single CP with structural flexibility, which facilitates the ease in genetic or chemical modifications of the VLPs or CVPs (1, 8). Many plant virus-derived VLPs or CVPs have been developed as vaccine candidates, such as the edible vaccine against rabies by incorporation of a recombinant peptide from the G and N proteins of rabies virus on the N-terminus of the alfalfa mosaic virus (AlMV) (4). Some of plant virus-derived vaccine candidates, including malaria (9) and influenza (10), have been tested in human clinical trials, demonstrating the safety and efficacy of such vaccines. In our previous studies, we have developed an efficient epitope presentation CVP system based on *Bamboo mosaic virus* (BaMV), a member of the genus *Potexvirus*, and validated its applicability in stimulating protective immunity against foot-and-mouth disease virus (FMDV) (11, 12), infectious bursal disease virus (IBDV) (13), or Japanese encephalitis virus (JEV) (14) in different animal models. However, these previously developed BaMV-based epitope presenting systems, as well as other VLP or CVP systems, still face several challenges that require further improvement.

One of the major limitations of the epitope-presenting VLP or CVP systems is that the fusion of target proteins on the viral coat proteins may impair the self-assembly efficiency of the VLPs or CVPs, which are required for highly ordered presentation of repetitive epitopes to stimulate strong immune responses. In addition, the fusion with the viral CP may interfere with the proper folding of the antigen proteins, leading to inactive conformation. Thus, the sizes of the target antigens to be displayed on VLPs or CVPs are usually limited to short peptide fragments. However, some epitopes are structurally-dependent, which require the formation of specific conformation from different domains of an antigen, not just linear amino acid sequences. The peptide epitopes might not stimulate proper immune responses. Several strategies have been developed to circumvent the above challenges. For example, we have adopted the FMDV 2A peptide sequence, which induces co-translational “cleavage” (15) between target epitope and CP, in our previous design for the production of BaMV-based vaccine candidate against JEV (14). Another approach is to attach the antigens after the assembly of the VLPs or CVPs, through chemical or enzymatic conjugation, such as sortase A (SrtA)-mediated ligation (16–18).

SrtA, an enzyme with both peptidase and ligase activities, was originally identified in *Staphylococcus aureus* and found to mediate the ligation of proteins on the surface of bacterial cells. It has been shown that different SrtA may recognize and cleave at specific amino acid sequences in the donor protein and mediate the ligation of the acceptor protein at the exposed N-terminus (19, 20). The coupling of target proteins on the surfaces of VLPs or CVPs by SrtA-mediated ligation has been successfully applied in different systems, including bacterial phage M13 (21) and papaya mosaic virus (PapMV) (17, 18, 22). However, these platforms could be further improved with regard to the convenience in the mass-production of VLP or CVP scaffolds, which is another major concern in the development of VLP or CVP antigen-presentation platforms.

To address these issues, we have devised a binary system in this study. The first part contains a BaMV-based vector which facilitates the efficient *in planta* production of CVPs self-assembled from CPs with 5 protruding glycine (G) residues as the acceptor for SrtA-mediated ligation (hereafter referred to as SrtA-ready CVPs). The second part includes vectors for the expression of target antigen, with calmodulin (CaM) fusion tag to enhance solubility, and SrtA protein, respectively, in bacterial cells. The applicability of this new system was demonstrated by using the recombinant envelope protein domain III (rEDIII) of JEV (111 amino acids) as the target epitope, and the efficacy of the vaccine candidate in the induction of functionalized antibodies was validated in BALB/c mice. The current BaMV-based system provides an efficient alternative for the rapid development of bioactive vaccine candidates.

## Methods

### Construction of Expression Vectors

**Figure 1:**
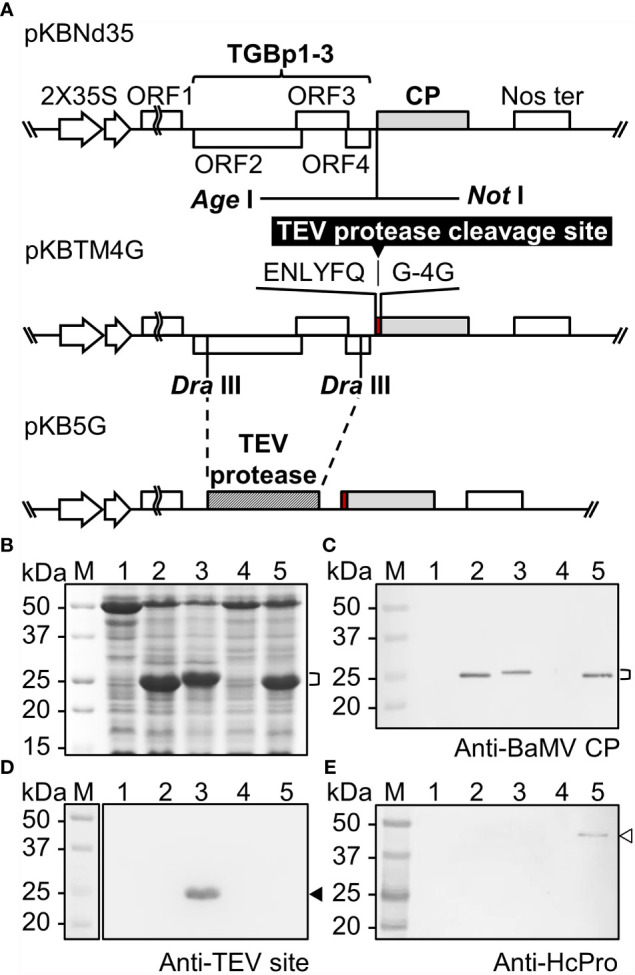
Production of SrtA-ready CVPs of BaMV in *Nicotiana benthamiana*. **(A)** Schematic representation of the construction of infectious clones for the production of SrtA-ready CVPs in *N. benthamiana* for SrtA-mediated ligation of antigens. **(B)** Analysis of total proteins extracted from *N. benthamiana* leaves infiltrated with *Agrobacterium tumefaciens* harboring individual constructs by electrophoresis through a 12% acrylamide gel containing 1% sodium dodecyl sulfate (12% SDS-PAGE), followed by staining with CBS. The positions of BaMV CP with various modifications were indicated by the bracket on the right. The proteins were then electro-blotted to PVDF membranes, and probed with antiserum specific to BaMV coat protein **(C)**, TEV protease recognition site **(D)**, or HC-Pro **(E)**. Total proteins extracted from *N. benthamiana* leaves infiltrated with buffer only (lane 1), or *A. tumefaciens* harboring pKBNd35 (lane 2), pKBTM4G (lane 3), or pKB5G (lane 4), or co-infiltrated with *A. tumefaciens* harboring pKB5G and pBIN-HC-Pro (lane 5); lane M, molecular weight markers. The positions of CP fused TEV site and HC-Pro are indicated by the solid and blank arrowheads on the right, respectively.

The constructs used for the production of BaMV CVPs were developed based on the infectious clone of BaMV, pBS-d35CP (11). To enhance viral vector expression, the original pBS-d35CP backbone was replaced by the binary plasmid pKn (23) to generate pKBNd35 (**Figure 1A**), which could be introduced into plant cells through *Agrobacterium*-mediated infiltration. The coding sequence for TEV protease cleavage motif (ENLYFQG, abbreviated as TM) and tetra-glycine (4G) were amplified by polymerase chain reaction (PCR) using the primer pair, TM-4G-F and TM-4G-R, and inserted into pBS-d35CP at the *Age*I/*Not*I sites, which was further digested with *ApaL*I and *Sac*I and inserted into the corresponding restriction sites of pKBNd35 to generate pKBTM4G. The coding region for BaMV triple-gene-block proteins (TGBps) was replaced by that of Histidine-tagged TEV protease (7×His-TEV protease), which was amplified by PCR using the primer pair, 7×His-TEV protease-F and TEV protease-stop-R, followed by digestion with *Dra*III and cloned into the corresponding site of pKBTM4G, generating pKB5G (**Figure 1A**). The pKB-based constructs were transformed into *Agrobacterium tumefaciens* GV3850 for subsequent CVP expression following confirmation of the sequence. Primer sequences for CVPs construction are listed in **Supplementary Table 1**.

**Figure 2:**
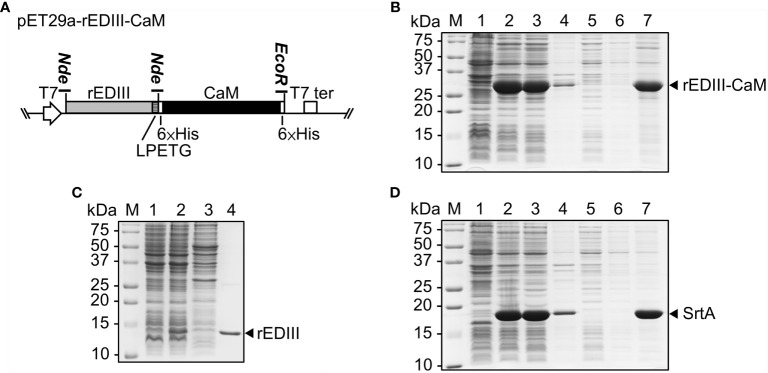
Production and purification of JEV rEDIII and SrtA in *Escherichia coli*. **(A)** Schematic of the construct, pET29a-rEDIII-CaM, for the expression of rEDIII fused with calmodulin (CaM) (rEDIII-CaM). The blank arrow and box represent the T7 promoter (T7) and T7 terminator (T7 ter), respectively. **(B–D)** SDS-PAGE analysis of bacterially expressed rEDIII, rEDIII-CaM and SrtA proteins. *E. coli* BL21 cells were transformed with pET29a-rEDIII-CaM **(B)**, pET21d-rEDIII **(C)**, or pET29-eSrtA **(D)**. Protein samples taken from the following steps or fractions were analyzed by 15% SDS-PAGE and visualized by CBS. Lanes 1, and 2, before and after IPTG induction, respectively; lanes 3 and 4, supernatant and pellet fraction, respectively, following sonication and centrifugation; lanes 5, 6, and 7, the flow-through, washing, and elution fractions, respectively, following purification by Ni^2+^-NTA affinity column; lane M, molecular weight markers. The positions of rEDIII (≈12 kDa), rEDIII-CaM (≈32 kDa), and SrtA (≈18 kDa) are indicated by the arrowheads on the right.

To improve the solubility of the recombinant proteins, we have employed CaM fusion tag, which has been used for proteins highly expressed in *E. coli* (24). The gene encoding CaM was amplified from *N. benthamiana* by using 6×His-CaM-F and CaM-GSS-6×His-R primers that incorporate 6×His tags at both termini of the main CaM coding sequence with *Nde*I and *Eco*RI sites. The amplified fragments of dual-6×His-tagged (6×His) CaM were digested with *Nde*I and *Eco*RI and ligated into the cognate sites of pET29a (Invitrogen, Waltham, MA, USA) to generate pET29a-6×His-CaM-6×His, abbreviated as pET29a-CaM. The coding sequence of JEV rEDIII was amplified from pBJ2A (14) with the primer pair, rEDIII-F and rEDIII-GSS-LPETG-GS-R, to add the SrtA recognition sequence, LPETG-GS, to the C-terminus of rEDIII. The amplified fragment was cloned into the *Nde*I site of pET29a-CaM to generate pET29a-rEDIII-CaM (**Figure 2A**), which harbors the coding sequence of rEDIII fused with the SrtA recognition site, LPETG-GS, followed by the coding region of CaM flanked by two His-tag (6×His) at both termini. We also attempted to use the common thioredoxin (TrxA) fusion tag, which was amplified from pET32a by using TGSS-TrxA-6×His-F and TrxA-8×His-R primers to replace CaM with TrxA on pET29a-CaM with *Age*I and *Eco*RI site to generate pET29a-6×His-TrxA-8×His, abbreviated as pET29a-TrxA. The coding sequence of VP1 peptide was amplified from pBVP1 (11) with the primer pair, VP1-F and VP1-GSS-LPETG-R, to add the SrtA recognition sequence, LPETG-GS, to the C-terminus of TrxA. The amplified fragment was cloned into the *Nde*I and *Age*I site of pET29a-TrxA to generate pET29a-VP1 peptide-TrxA (**Supplementary Figure 8**). Primer sequences for rEDIII-CaM and VP1 peptide-TrxA constructions are listed in **Supplementary Table 1**. For production of SrtA, the plasmid pET29-eSrtA was purchased from Addgene (Watertown, MA, USA). The pET29-based expression constructs were transformed into the *E. coli* BL21 Star (DE3).

### Protein Expression and Purification

Following cultivation of *E. coli* BL21 Star (DE3) cells harboring the desired constructs, the induction and purification of target proteins were performed as described previously (14). The His-tagged target proteins were subjected to purification through a Ni^2+^-NTA column (GE Healthcare, Chicago, IL, USA). The fractions containing rEDIII-CaM and VP1 peptide-TrxA proteins were confirmed by 12% polyacrylamide gel containing 1% sodium dodecyl sulfate (12% SDS-PAGE). The rEDIII protein, which was expressed from pET21d-rEDIII (14), was produced and purified as described previously (14, 25).

### BaMV CVP Production

The pKB-based clones and pBIN61-HC-Pro (26) were introduced into *N. benthamiana* plants through *Agrobacterium*-mediated infiltration (27) with the concentrations of *A. tumefaciens* cells harboring pKBNd35, pKBTM4G, pKB5G, or pBIN61-HC-Pro adjusted to an OD_600_ of 0.5. For co-infiltration, equal volumes of *A. tumefaciens* cells harboring pKB5G or pBIN61-HC-Pro were mixed and adjusted to make a final concentration of OD_600_ = 0.5 for each. The infiltrated leaves were harvested 7 days post-inoculation (DPI), and BaMV CVPs were purified using the standard protocol described previously (28).

### Production of Epitopes-Coupled CVPs

The CP concentration of BaMV CVP was adjusted to 1 mg/mL and subject to SrtA-mediated ligation reaction. The ratio between the volumes of BaMV CVP and the total reaction mixture was adjusted to 1:10. To determine the optimal reaction condition of the rEDIII coupling to BaMV CVPs, the ratio of B5G CP: rEDIII-CaM: SrtA was varied as indicated in a 1.5-ml Eppendorf tube containing 1× SrtA reaction buffer (50 mM Tris-HCl, pH 8.0, 150 mM NaCl, 10 mM CaCl_2_, and 4 mM beta-mercaptoethanol), and the reaction mixture was incubated at 28°C for different time period as indicated. SrtA activity was terminated with the addition of 0.25 reaction volume of 5× sample buffer (250 mM Tris-HCl, pH 6.8, 10% SDS, 40% (v/v) glycerol, 2 mM beta-mercaptoethanol, 0.1% (w/v) bromophenol blue). The protein products were analyzed by electrophoresis through 12% SDS-PAGE, followed by Coomassie Blue Staining (CBS) or western blotting with specific antibodies as described below. Large-scale coupling reactions were performed in a 100-ml beaker, and the mixture was incubated for 15 min at 28°C with gentle stirring. Another epitope candidate, the VP1 peptide-presenting BaMV CVPs (designated BVP1LPET5G) were also produced as described above.

### Purification of Epitopes-Coupled BaMV CVPs

The rEDIII-presenting BaMV CVPs (designated BJLPET5G) were purified as described above with the following modifications: (1) the chelator (10 mM EGTA) and disodium phosphonate (100 mM K_2_HPO_3_) were gently added to inhibit SrtA activity; (2) the calcium complex was removed by centrifugation at 7,600 ×*g* at 4°C for 10 min; (3) the supernatant was purified with the Ni^2+^-NTA column to remove the unreacted His-tagged proteins; (4) the flow-through fraction was collected and subjected to ultracentrifugation through a 20% sucrose cushion; and (5) the virion pellet was resuspended with Borate–EDTA buffer (50 mM boric acid, pH 8.0, and 1 mM EDTA) and stored at 4°C. The BVP1LPET5G CVPs were inhibited SrtA activity as described above and purified using the PEG 6000 precipitation protocol as described previously (28).

### Protein Analysis and Antibodies

Total proteins were extracted from infected *N. benthamiana* leaves and/or *E. coli* cultures, and subjected to analysis by SDS-PAGE and western blot (29) using specific antibodies against BaMV CP (5000× dilution), HC-Pro (5000× dilution), JEV rEDIII (5000× dilution), FMDV VP1 (5000× dilution), or commercial polyclonal antibodies against TEV protease recognition site (Abcam, Cambridge, United Kingdom; 2500× dilution), or mouse monoclonal antibody against His-tag (Bio-Rad, Hercules, CA, USA; 2000× dilution), followed by visualization using a nitro-blue tetrazolium/5-bromo-4-chloro-3’-indolyphosphate (NBT/BCIP) color development substrate (Thermo Scientific, Waltham, MA, USA) or horseradish peroxidase (HRP) reagent (Merck Millipore, Darmstadt, Germany). The Precision Plus Protein Dual Color standard marker (Bio-Rad, Hercules, CA, USA) was used as the molecular weight standard.

### Quantification of CVPs and Proteins

The amount of CVPs was determined spectrophotometrically as described previously (28). The concentrations of rEDIII-CaM, SrtA, and rEDIII proteins were determined through the Bradford assay (Sigma-Aldrich, St. Louis, MO, USA). To quantify the concentration of rEDIII proteins on the CVPs, SrtA was used to release the rEDIII coupled on CVPs and the digested products were subjected to analysis by 12% SDS-PAGE followed by staining with CB and quantification. Known amounts of rEDIII were used to create a standard for quantification using ImageJ software (30).

### Immunoelectron Microscopy

Negative staining and immunoelectron microscopy with gold-labeled antibodies specific to the BaMV virion or JEV rEDIII were performed as described previously (31). CVP conformation and immunolabeling were examined through transmission electron microscopy (FEI Tecnai G2 Spirit; FEI Company, Hillsboro, OR, USA) at 80 kV. The control grids were loaded with BNd35 and B5G CVPs.

### Animals and Vaccination

Three groups of five 3-week-old female BALB/c mice were obtained from the National Laboratory Animal Center, Taiwan. Freund’s complete (priming) or incomplete adjuvants (boosting) (Sigma-Aldrich, St. Louis, MO, USA) were mixed with B5G (BaMV virion scaffold), rEDIII (5 μg), and BJLPET5G (≈5 μg of rEDIII-coupled BaMV virion scaffold) for priming on day 1 and boosting on day 12, respectively, for intraperitoneal inoculation. Whole blood from the orbital sinus was collected on days 0, 11, and 26; the blood cells were removed through centrifugation (13,000 ×*g*, 10 min) twice, and the sera were collected. The titers of sera were tested through indirect enzyme-linked immunosorbent assay (ELISA).

### Mice Immunization Assay

C6/36 cells were infected with JEV (RP-9) (32) at a multiplicity of infection (MOI) of 0.1 and cultured for 4 days in a 96-well plate. The infected cells were fixed and examined by indirect ELISA with diluted serum samples collected from immunized mice overnight at 4°C followed by detection with goat anti-mouse IgG-HRP conjugate (Jackson ImmunoResearch Laboratories, West Grove, PA, USA) as described previously (33). The OD_450_ was measured by using an ELISA reader (Spectramax M2; Molecular Devices, Sunnyvale, CA, USA).

### Immunofluorescence Assay

BHK-21 cells were infected with the reporter virus, JEV (RP-9) expressed enhanced green fluorescent protein (JEV-eGFP) (34), at an MOI of 2 for 15 h. Following fixation and permeabilization, the cells were incubated overnight at 4°C with a serial dilution of different sera from mice vaccinated with B5G, rEDIII, or BJLPET5G followed by detection with goat anti-mouse IgG-Alexa 568 conjugate (Invitrogen, Waltham, MA, USA), and examined under a fluorescence microscope. Fluorescent signal images were captured at 200× magnification with an Olympus IX71 inverted fluorescence microscope (Olympus, Tokyo, Japan).

### Reporter Virus Neutralization Test

The titers of the neutralizing antibodies in the sera from mice vaccinated with various CVPs were determined using the neutralization assay with BHK-21 cells and the reporter virus JEV-eGFP (at an MOI of 0.5) in triplicates following the standard protocol (35). The JEV-eGFP was incubated with sera from vaccinated mice with 2.5-fold serial dilutions (starting at 1:40) in Opti-MEM medium (Gibco, Grand Island, NY, USA) at 37°C for 1 h. The mixtures were individually incubated with a monolayer of BHK-21 cells in the 96-well plates at 37°C for 2 h. After incubation, the inoculum in the plates was replaced with fresh medium containing 2.5% FBS for incubation at 37°C for 20 h. After fixation and permeabilization, the nuclei were stained with 4′, 6-diamidino-2-phenylindole (DAPI; Invitrogen, Waltham, MA, USA). Finally, the plates were scanned under a fluorescence microscope to generate the panels, and the cells were scored (percentage of infectivity = fluorescent cells/total cells) using MetaXpress software (Molecular Devices, San Jose, CA, USA) for high content screening. The 70%-neutralization titer in RVNT (RVNT_70_) data for each antibody were used to calculate relative reduction in fluorescence of JEV-eGFP, determined as follows: % reduction = 100 × [1 − (average number of fluorescent cells for each dilution/average number of fluorescent cells in B5G control group)]. The RVNT_70_ is evaluated by the concentration that gave 70% fluorescent cells reduction, determined by nonlinear, dose-response regression analysis with GraphPad Prism version 6.0 (GraphPad, San Diego, CA, USA).

### Streamlined Production of Epitope-Presenting CVPs

His-tagged rEDIII-CaM and SrtA proteins were purified from bacterial cell lysates (following sonication) with Ni^2+^-NTA resin in batch mode, and bound on resin for the coupling reaction with SrtA-ready B5G CVPs. Mass-produced SrtA-ready B5G CVPs were added to the resin with rEDIII-CaM and SrtA, incubated for 15 min in the SrtA reaction buffer containing 300 mM imidazole. The SrtA-mediated ligation reaction was terminated with the addition of EGTA (10 mM), and the imidazole was removed by using spin column (Amicon Ultra 10K, Merck Millipore). Supernatant was incubated with Ni^2+^-NTA resin again to remove his-tagged unreacted proteins. The BJLPET5G CVPs were then harvested from the supernatant after low-speed centrifugation.

### Statistical Analysis

Statistical analysis and graphical visualizations were performed in Microsoft Excel 2010 (Microsoft, Redmond, WA, USA) and GraphPad Prism version 6.0. A two-tailed Student’s *t* test was used to compare between the means of two groups; *p* < 0.05 indicates statistical significance.

## Results

### Production of SrtA-Ready Chimeric BaMV Particle *In Planta*

In this study, we have developed a CVP scaffold based on BaMV that is ready for the presentation of target protein through SrtA-mediated ligation, as shown in **Figure 1A**. This design ensured the production BaMV CP and TEV protease within the same plant cell. The TEV protease could thus recognize the TM at the N-terminus of the modified BaMV CP and cleave in between Q and G, generating BaMV CP with 5 protruding G residues which could then self-assemble into BaMV CVPs, designated as B5G, susceptible for SrtA-mediated ligation of target proteins. The assembled BaMV CVPs are then equipped with thousands of protruding 4G extensions ready for the SrtA-mediated ligation of the antigens. The above constructs were then used to inoculate *Nicotiana benthamiana via Agrobacterium*-mediated infiltration. Total proteins were extracted from the inoculated leaves 7 DPI and analyzed by electrophoresis through 12% SDS-PAGE, followed by CBS (**Figure 1B**) and western blot analysis using specific antiserum (**Figures 1C–E**). As shown in **Figures 1B, C**, BaMV CP could be detected in total protein extracts from *N. benthamiana* leaves inoculated with *A. tumefaciens* harboring pKBNd35 and pKBTM4G (lanes 2 and 3, respectively), but not pKB5G (lane 4). We hypothesized that the low expression level might be due to the replacement of the original BaMV RNA silencing suppressor, TGBp1, by the TEV protease in pKB5G construct, leading to the silencing of the expressions of B5G CP. To address this problem, we resorted to the usage of the well-known potyvirus silencing suppressor, HC-Pro (36). *A. tumefaciens* cells harboring pKB5G were co-infiltrated with those harboring pBIN61-HC-Pro (26) to complement the silencing suppressor function of BaMV TGBp1. As expected, the co-expression of potyviral HC-Pro facilitated the efficient accumulation of B5G proteins in the infiltrated leaves (**Figures 1B, C**, lane 5). To determine whether the TM on BaMV CPs is functional in plants, the total proteins extracted from infiltrated tissues were probed using antibodies specific to TM (**Figure 1D**). The result showed that only the BaMV CP from leaves inoculated by pKBTM4G could be detected (**Figure 1D**, lane 3), indicating that the TM could indeed be recognized and digested by the TEV protease expressed in the same plant cell infiltrated with pKB5G, rendering it undetectable by the TM-specific antibodies (**Figure 1D**, lane 5). The higher mobility of B5G (**Figures 1B, C**, lane 5) compared to that of the CP from leaves infiltrated with pKBTM4G (**Figures 1B, C**, lane 3) provide further evidence indicating that the TM on the B5G was digested to a similar size of BNd35 CP (**Figures 1B, C**, lane 2) by TEV protease. The presence of HC-Pro in the leaves co-infiltrated with pKB5G and pBIN61-HC-Pro was confirmed by HC-Pro-specific antibodies (**Figure 1E**, lane 5).

### Protein Expression and Purification in *Escherichia coli*

To prepare the donor protein, rEDIII, to be presented on the surface of B5G CVPs, we have developed a vector, designated pET29a-rEDIII-CaM (**Figure 2A**), for the expression of target proteins in *E. coli* with the C-terminal fusion of CaM. The protein product expected to be translated from such construct would be rEDIII-LPETG-GS-6×His-CaM-6×His, abbreviated as rEDIII-CaM, in which the rEDIII-LPET fragment could be attached to the G residue of the acceptor protein by SrtA-mediated ligation and the released G-GS-6×His-CaM-6×His fragment could be removed by Ni^2+^-NTA affinity column. To test the functionality, the construct pET29a-rEDIII-CaM was transformed into *E. coli*, and the target protein expression was induced by the addition of IPTG. As shown in **Figure 2B**, the expected fusion protein rEDIII-CaM (lane 2) was successfully expressed and detected by the previously prepared JEV-specific antibodies (**Supplementary Figures 1A, B**, lane 4). In comparison with the rEDIII expression system without CaM-fusion, the level of soluble rEDIII protein in *E. coli* cells expressing CaM-fused rEDIII was increased in the supernatant, resulting in the highest amount of total rEDIII protein extracted after sonication (**Figures 2B, C**, lanes 3 and 4).

To provide SrtA proteins for ligation reactions, the construct pET29-eSrtA purchased from Addgene (19), which harbors the coding sequence of SrtA with 6×His tag, was transformed into *E. coli*. Following IPTG induction, the SrtA proteins could be readily detected in the total protein extracts by 15% SDS-PAGE through CBS (**Figure 2D**, lane 2). Both the 6×His-tagged rEDIII-CaM and SrtA could be easily purified by Ni^2+^-NTA resin (**Figures 2B, D**, lanes 5–7), and the protein samples were stored in 50% glycerol at −20°C for subsequent SrtA-mediated ligation reactions.

### SrtA-Mediated Surface Modification on B5G CVPs

**Figure 3:**
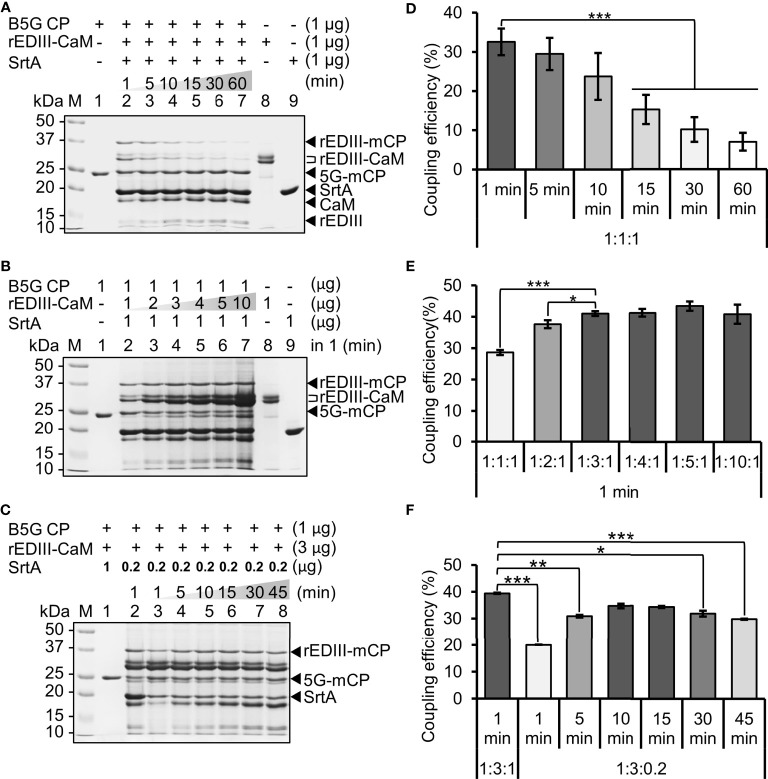
Condition optimization of SrtA-mediated ligation of rEDIII on the B5G CVP surface. **(A)** Effect of reaction time. Equal amounts of B5G CP, rEDIII-CaM, and SrtA were incubated for different reaction times as indicated on top of each lane. **(B)** Effect of different ratios of reactants. The amount of rEDIII-CaM was increased by 2 to 10 folds, as indicated on top of each lane, relative to that of B5G CP, with a reaction time of 1 min. **(C)** Effect of reaction time with reduced SrtA concentration. The amount of SrtA was reduced to 0.2 μg and the reaction time was varied as indicated on top of each lane. The components in each reaction are shown on top of each lane. Protein samples were analyzed by 12% SDS-PAGE, and the protein bands were visualized by CBS. The expected sizes of different forms of proteins were indicated by the arrowheads and brackets on the right. The relative coupling efficiencies under different reaction conditions were shown in the respective bar charts **(D–F)**. Statistical significance was analyzed using two-tailed Student’s *t* tests; *p < 0.05; **p < 0.01; ***p < 0.001. Error bars represent the standard deviation of three independent experiments.

To evaluate the feasibility of SrtA-mediated coupling on the B5G viral surface, small-scale reactions were performed using equal amounts of B5G CP, rEDIII-CaM, and SrtA. Time-course assays were then performed to test the optimal reaction time to maximize the yield of epitope-presenting BaMV CVPs. The LPETG-GS motifs on rEDIII-CaM were cleaved and ligated on B5G CPs by SrtA, resulting in the generation of rEDIII-LPET-5G-mCP (abbreviated as rEDIII-mCP) with a size of approximately 35 kDa, as observed by SDS-PAGE analysis (**Figure 3A**). The result revealed that the SrtA-mediated coupling of rEDIII on BaMV CVPs could be achieved in 1 min (**Figure 3A**), generating rEDIII-presenting BaMV CVPs (designated BJLPET5G). The identities of the coupled proteins and rEDIII proteins were further confirmed by western blot analysis with specific antibodies against BaMV CP or rEDIII (**Supplementary Figure 2**). To evaluate the relative coupling efficiency, we calculated the ratio between the band intensities of the rEDIII-coupled CPs and the total CPs in SDS-PAGE (**Figures 3A–C**) using the software ImageJ (National Institutes of Health, Bethesda, MD, USA) (**Figures 3D–F**). It was found that up to 35% of the B5G CPs were labeled with rEDIII by SrtA in 1–10 min, whereas only 3% to 18% of B5G CPs retained the rEDIII after 15 min (**Figures 3A, D**). Longer reaction time (more than 15 min) resulted in significantly decreased yields of BJLPET5G, presumably caused by the removal of rEDIII from BaMV CVPs by SrtA, since the ligation of the rEDIII on the B5G CVPs would create another SrtA-recognition site which could be cleaved again in a prolonged reaction (37, 38). With this knowledge of the SrtA reaction time, the reaction condition was optimized by increasing the amount of rEDIII-CaM from 2 to 10 folds relative to that of B5G CP to increase the amount of rEDIII protein labeled on the B5G CVPs. The result showed that the amount of conjugated rEDIII on the CVPs reached a plateau when the ratio between rEDIII-CaM and B5G CP was increased to 3:1 or more (**Figures 3B, E**). To avoid the removal of rEDIII following SrtA-mediated ligation, the ratio between B5G CP and SrtA was decreased to 1:0.2 in the subsequent time-course experiment. It was found that similar coupling efficiency could be observed with longer reaction times (10-15 min), whereas the labeled rEDIII proteins were re-cleaved after 30 min (**Figures 3C, F**). Accordingly, the reaction time was extended in the following experiments to increase coupling efficiency and facilitate subsequent purification. The coupled proteins on the CVPs were then quantified following SDS-PAGE with the software ImageJ. It was found that the coupling efficiency with the rEDIII protein was approximately 22.5-43.0% (**Figures 3C, F**). Based on these observations, the optimal ratio for coupling was determined to be 1:3:0.2 for B5G CP, rEDIII-CaM, and SrtA, with the corresponding concentrations of approximately 4.38, 9.48, and 1.12 μM, respectively.

To demonstrate the applicability of the B5G CVP platform with SrtA-mediated ligation for other antigens, we used the VP1 peptide derived from FMDV (14) as the target protein. The result showed that the VP1 peptide could also be coupled to B5G CP at a ratio of 1:2:0.2 for B5G CP, VP1-TrxA, and SrtA, with an efficiency up to 42% (**Supplementary Figure 9**).

### Optimization of Purification Process and Quantification of SrtA-Coupled CVPs

**Figure 4:**
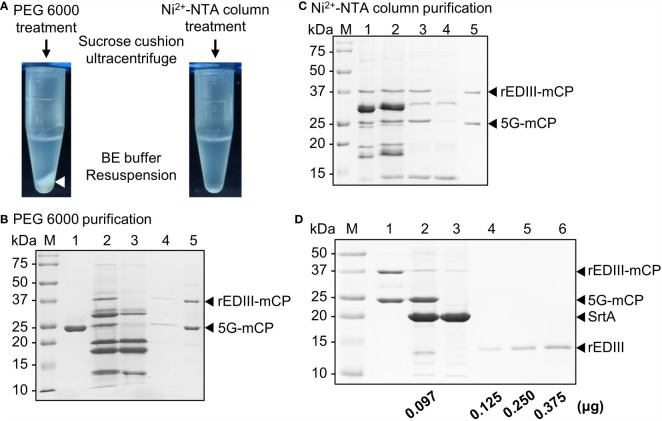
Purification and quantification of B5G CVPs presenting rEDIII. **(A)** Comparison of purification by polyethylene glycol (PEG) 6000 precipitation (left) or Ni^2+^-NTA affinity column (right). The BaMV CVPs presenting rEDIII following SrtA-mediated ligation were separated from the unreacted rEDIII and SrtA by an initial purification of PEG 6000 precipitation or Ni^2+^-NTA affinity column, followed by ultracentrifugation through a 20% (w/v) sucrose cushion. For the initial separation using PEG 6000, white precipitate was observed (white arrowhead). **(B)** SDS-PAGE analysis of BJLPET5G CVPs purified by PEG precipitation. Lanes 1, B5G alone; 2, unpurified SrtA reaction mixture; 3, supernatant collected after PEG precipitation. The pellet fraction obtained following PEG precipitation was resuspended and subjected to ultracentrifugation, and the resulting supernatant (lane 4) and final pellet (lane 5) fractions were analyzed. **(C)** SDS-PAGE analysis of BJLPET5G CVPs purified through Ni^2+^-NTA affinity column. Lanes 1, unpurified SrtA reaction mixture; 2, reaction mixture containing EGTA and K_2_HPO_4_ for inhibiting SrtA activity; 3, flow-through sample from Ni^2+^-NTA column; 4 and 5, the supernatant and pellet fraction, respectively, obtained following ultracentrifugation through a 20% sucrose cushion. **(D)** SDS-PAGE analysis of the amount of the rEDIII proteins coupled on the CVP surface. Purified BJLPET5G CP (2 μg) were cyclically digested by SrtA (4 μg) at 28°C overnight to release the rEDIII. The digestion product was analyzed by 12% SDS-PAGE and visualized by CBS. Lanes M, molecular weight standard; 1, undigested BJLPET5G CVPs; 2, digested BJLPET5G CVPs; 3, SrtA protein control; 4-6, increasing amounts (0.125–0.375 μg) of purified rEDIII from *E. coli* as the quantitative standard. The relative amount of coupled rEDIII on the CVPs was quantified by analyzing the band intensities using ImageJ, as indicated at the bottom. The positions of various proteins were indicated by arrowheads on the right.

Following the addition of 10 mM ethylene glycol tetraacetic acid (EGTA) to terminate SrtA reaction, the virions were extracted from the mixture as described (28). Unexpectedly, the polyethylene glycol (PEG)-mediated CVPs precipitation resulted in the generation of insoluble white precipitates (**Figure 4A**, left panel). Analysis by SDS-PAGE revealed that most of the free CPs or rEDIII-coupled CPs were detected in the pellet fractions (either before or after sucrose cushion purification), indicating that PEG treatment severely reduced the solubility of CVPs (**Figure 4B**, compare lanes 4 and 5). To enhance the solubility of the final product, we employed affinity column purification using Ni^2+^-NTA resin. It was found that the affinity purification method prevented the precipitation problem (**Figure 4A**, right panel). The result of SDS-PAGE analysis revealed that most bands corresponding to CP with or without the rEDIII labels were harvested in the flow-through fraction (**Figure 4C**; lane 3, indicated by arrowheads). In addition, most CPs could be easily separated from free SrtA and rEDIII-CaM by ultracentrifugation through a 20% sucrose cushion, suggesting that CPs were assembled into BJLPET5G CVPs (**Figure 4C**, lane 5). The recovery rate of CVP CP was approximately 82-98%, as determined from the band intensities in SDS-PAGE (**Figure 4C**, lanes 1 and 5, respectively). To evaluate the amount of coupled rEDIII on the CVP CP, SrtA-mediated cyclic cleavage was performed to release the coupled rEDIII, and the products were analyzed by SDS-PAGE (**Figure 4D**). The result of quantification using software ImageJ revealed that approximately 97 ng rEDIII were coupled on 1 μg of B5G CP. As indicated by the structural model of the BaMV virion, each CP unit interacts with estimated 5.2 bases (39). Based on this calculation and the observed coupling efficiency, the estimated numbers of BJLPET5G CPs per CVP are approximately 345 out of 1150 copies.

### Identification of rEDIII on the BJLPET5G Surface Through Immunoelectron Microscopy

**Figure 5:**
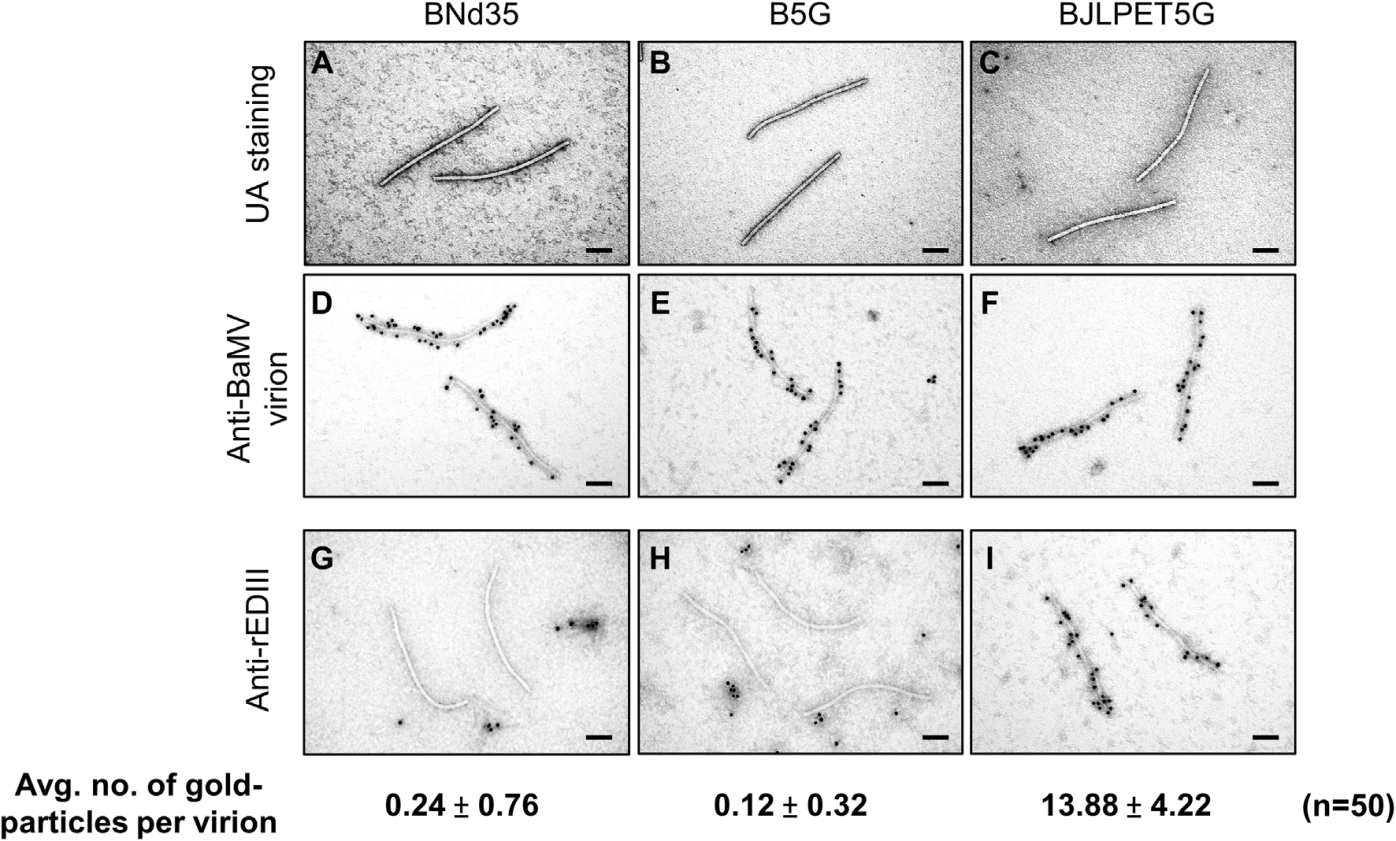
Examination of BaMV CVPs through transmission electron microscopy. Purified CVPs of BNd35 **(A, D**, **G)**, B5G **(B, E**, **H)**, and BJLPET5G **(C, F**, **I)** were observed by transmission electron microscopy using uranyl acetate (UA) negative staining **(A–C)**, or immuno-gold staining using antisera specific to BaMV virion **(D–F)** or JEV rEDIII **(G–I)** followed by labeling with the gold-conjugated goat anti-rabbit IgG antibodies. Average numbers of gold particles on each CVP surface (n = 50) treated with antisera-specific rEDIII **(G–I)** are indicated below each panel. Scale bars, 100 nm.

To verify whether the CVPs of B5G and BJLPET5G were properly assembled, the purified samples were examined by electron microscopy, with the BNd35 virion as a control. The result showed that the CVPs of B5G and BJLPET5G exhibited similar flexible filamentous virion conformations as those of BNd35 (**Figures 5A–C**, respectively). Immunoelectron microscopy was performed to confirm the presence of rEDIII on the surface of BJLPET5G virions. As expected, BNd35, B5G, and BJLPET5G virions could be specifically decorated with gold particles following the reaction with antibodies against BaMV virions and gold-labeled secondary antibodies (**Figures 5D–F**, respectively). The presence of rEDIII on the surface of BJLPET5G virion scaffolds was verified by antibodies specific to rEDIII and gold-conjugated secondary antibodies, whereas little or no gold particles were observed on the BNd35 or B5G virions following the same treatment (**Figures 5G–I**). Quantification of the gold particles decorating virion surface revealed that each BJLPET5G virion was labeled by approximately 14 gold particles (an average of 50 virions, **Figure 5** and **Supplementary Figures 3A–C**). Compared to our previous BaMV-based vector with FMDV 2A peptide-fusion [BJ2A CVPs, (14)], it was found that BJLPET5G particles exhibited a 7.5-fold increase of rEDIII on the CVP surface (**Supplementary Figures 3C, D**). The result confirmed that rEDIII was successfully presented on the BJLPET5G virion surface.

### BJLPET5G Elicited Specific Antibody Responses and Virus-Neutralization in Mice

**Figure 6:**
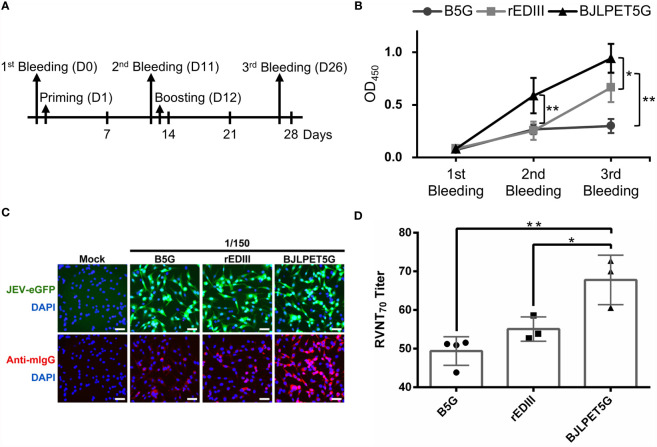
Immunization and virus-neutralization analysis. **(A)** Vaccination and bleeding scheme. The bleeding and injection date are indicated by the arrows. **(B)** The serum collected at each stage was diluted 500-fold, incubated with JEV-infected (RP-9) C6/36 cells coated on plates, and analyzed for antibody titers in an enzyme-linked immunosorbent assay (ELISA). The line chart represents the mean values of optical density at 450 nm (OD_450_) obtained in the ELISA with sera from individual mouse. **(C)** In the immunofluorescence assay, BHK-21 cells infected with JEV-eGFP (green) were fixed and stained with pooled serum diluted 150-fold from the immunized groups of mice. The antibodies were labeled with Alexa Fluor 568-conjugated secondary antibody against mice IgG (anti-mIgG, red), and the cell nuclei were stained with DAPI (blue). The samples were examined for antibody labeling by using an inverted fluorescence microscope. Scale bars, 50 μm. **(D)** The 70%-neutralizing antibody titrations of reporter virus neutralization test (RVNT_70_) against JEV-eGFP were preformed using serum samples collected in third bleeding from B5G, rEDIII, and BJLPET5G-vaccinated mice (n = 5). Individual samples were serially diluted from 1:40 to 1:625 and RVNT_70_ were performed in 96-well plates as described in material and methods. Sera from mice immunized with B5G were used as negative controls. rEDIII was used as the positive control. Approximately 5 μg of rEDIII was used for immunization in both the rEDIII and BJLPET5G groups. The relative mean and standard deviation of titers were shown in the bar chart. The standard deviations are indicated by the error bars. Statistical significance was analyzed using two-tailed Student’s *t* tests; *p < 0.05; **p < 0.01.

To assess the immunogenicity of BJLPET5G CVPs, we injected BALB/c mice (5 per group) with B5G (52.25 µg), free rEDIII (5 µg), or BJLPET5G (corresponding to 5 µg of rEDIII conjugated on 52.25 µg of B5G CP) following the scheme illustrated in **Figure 6A**. It was found that the antibody titers against JEV in serum samples collected from mice injected with BJLPET5G were increased more rapidly following the priming injection, as compared with the other groups (**Figure 6B**). After boosting, the JEV-specific antibody titers in the sera of mice in the BJLPET5G group have increased significantly as compared with those in the B5G and rEDIII groups (**Figure 6B** and **Supplementary Figure 4**). To compare the sera titers of different treatment groups, the titration curves for different time-points (i.e., pre-immune, priming, boosting) were plotted as shown in **Supplementary Figure 5**. In the analysis, the half-maximal effective concentration (EC_50_) values could not be accurately determined since the reaction curves had not reached a plateau at 1/100 dilution in our experiments. The observation indicated that immune responses elicited by the treatments were not especially strong. However, the data indicated that BJLPET5G stimulated higher or comparable titers than rEDIII did at the priming and boosting stages, respectively. In addition, the activities of antibodies were evaluated by using the JEV-eGFP reporter system (34), in which the cultured BHK-21 cells infected with JEV-eGFP were fixed and reacted with antisera (150-fold dilution) collected from mice in the aforementioned groups, followed by the detection of the bound antibodies by Alexa Fluor 568-conjugated secondary antibodies against mice IgG. The numbers of green fluorescence-positive cells were used as an indicator of infection, while the intensity of the red fluorescence represented the titer of the rEDIII-specific antibodies present in the serum samples. The result of fluorescence microscopy examination revealed that while the BHK-21 cells were infected with relatively equal amounts of JEV-eGFP (**Figure 6C**, upper panel), the sera from mice in the BJLPET5G group exhibited the highest titer against JEV, giving the highest intensities of red fluorescence as compared to those from mice in the B5G or rEDIII groups (**Figure 6C**, lower panel). This observation demonstrated that injection of BJLPET5G in mice could stimulate effective immune response, leading to the production of antibodies against JEV. To further verify whether the injection of BJLPET5G could induce the generation of neutralizing antibodies against JEV, we performed a RVNT, which is correlated with immune protection (34, 35). The result showed that JEV-eGFP pre-incubated with sera from mice in the BJLPET5G group resulted in fewer JEV-positive cells, as compared with those treated with sera from mice in the B5G or rEDIII groups (**Supplementary Figure 6**). The result indicated that sera from mice in the BJLPET5G group had a significantly superior ability in suppressing JEV infection compared to those from mice in the rEDIII group.

To further quantitate the JEV-inhibition efficiency, the pooled sera were serially diluted, and the inhibition rate was determined by the RVNT_70_ (14, 35). The result showed that the JEV-specific neutralizing antibody titers of the BJLPET5G group were significantly higher (**Figure 6D**), demonstrating the immunogenicity of BJLPET5G against JEV.

### Streamlined Production of Epitope-Presenting CVPs

**Figure 7:**
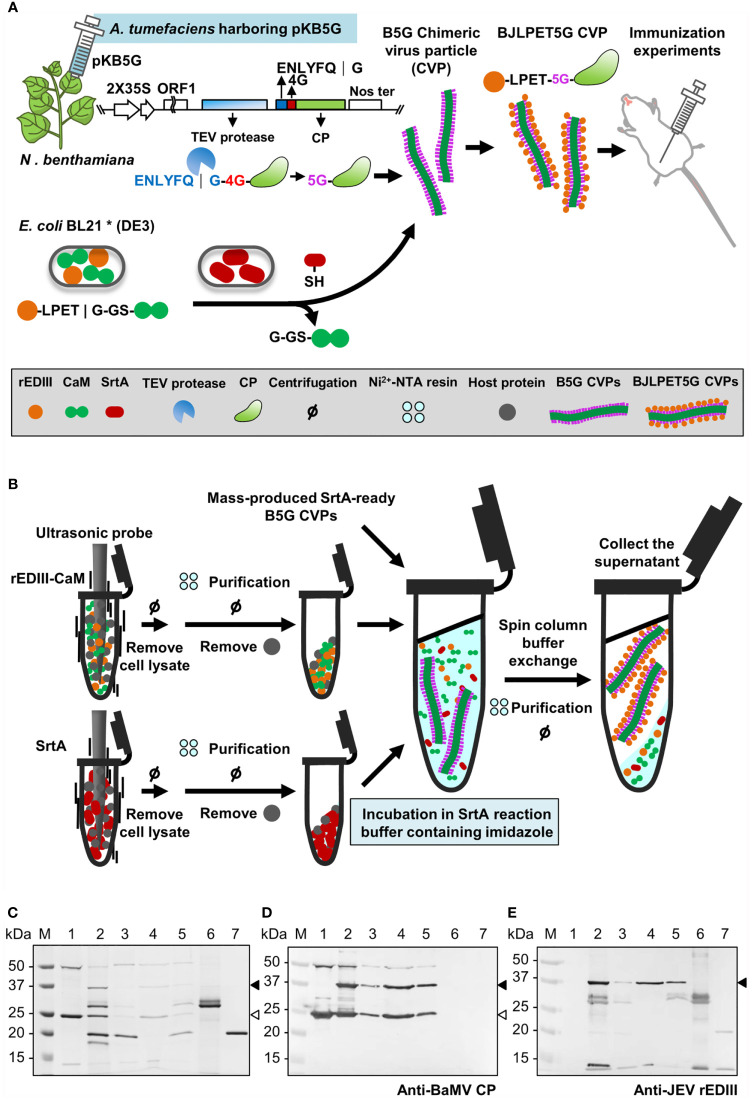
Illustration of the processes for the production of antigen-presenting CVPs. **(A)** Schematic representation of BaMV B5G-based SrtA-mediated CVP platform for JEV vaccine. The identities of the icons are indicated in the box at bottom. **(B)** Schematic representation of the simplified production process of epitope-presenting CVPs as new vaccine candidates. **(C–E)** SDS-PAGE and western blot analysis of BJLPET5G CVPs purified through Ni^2+^-NTA batch purification. The proteins were separated by 12% SDS-PAGE, visualized by CBS **(C)**, or electro-blotted to PVDF membranes and probed with antiserum specific to BaMV coat protein **(D)** or JEV rEDIII protein **(E)**. Lanes 1, unpurified SrtA reaction mixture; lanes 2 and 3, the supernatant and pellet fraction, respectively, obtained through a low-speed centrifuge after EGTA treatment; lanes 4 and 5, the supernatant and pellet fraction, respectively, obtained following buffer exchange, Ni^2+^-NTA resin incubation, and a low-speed centrifugation. Purified rEDIII-CaM (lane 6) and SrtA (lane 7) proteins were used as size markers. The positions of rEDIII-mCP and 5G-mCP are indicated by the solid and blank arrowheads on the right, respectively.

The workflow of the binary system is illustrated in **Figure 7A**. To demonstrate the applicability of the system in the efficient development of new vaccine candidates, we have tested the feasibility of a simplified protocol as follows (**Figure 7B**). The *E. coli* cells expressing the epitope (rEDIII-CaM in this example) and SrtA proteins were lysed by ultrasonication, and the lysates were directly mixed with Ni^2+^-NTA resin in the Eppendorf tube followed by the addition of previously prepared SrtA-ready B5G CVPs. The supernatant containing BJLPET5G CVPs was then recovered. However, the result of SDS-PAGE analysis of the products (**Figure 7C**) showed that the activity of SrtA-mediated ligation reaction was inhibited when SrtA proteins bound on the Ni^2+^-NTA resin. Thus, a modification of the above protocol was made in which the imidazole was added to release SrtA proteins (**Figure 7B**). Analysis of the final products by SDS-PAGE and western blot revealed that BJLPET5G CVPs could be readily obtained through this simplified protocol within 6 hrs (**Figures 7C–E**, lane 4). The result suggested that the system developed in this study could be applied in the rapid development of new vaccine candidates.

## Discussion

### Development of a Binary System for the Production of SrtA-Ready CVP Scaffolds and Antigens

The design of B5G CVP system ensured the production of TEV protease and BaMV CP within the same cell, and the processed BaMV CP could auto-assemble into the SrtA-ready virions *in planta*. In addition, the yield of B5G virions, reached about 1.7 mg per 100 g of fresh *N. benthamiana* leaves, with the co-expression of HC-Pro, which is comparable to the yields of wild type BaMV virions or CVPs in inoculated leaves or transgenic plant cell‐suspension system (11, 40). For the target antigen production, the fusion of 6×His-CaM-6×His tag at the C-terminus following the SrtA recognition site of the target antigen increased the solubility (**Figures 2B, C**) and allowed for easy purification of the target protein. By separating the production processes of CVPs and target antigens, this approach exhibits the potential to facilitated the auto-assembly of filamentous CVP scaffolds and the presentation of large peptides on the surface of such CVP scaffolds through SrtA-mediated ligation. We have showed the successful coupling of two different antigens, rEDIII and VP1 peptides, with B5G CVPs (**Figure 4** and **Supplementary Figure 9**), demonstrating the applicability of the current system for antigen presentation.

Compared to our previous design (14), the system developed in this study exhibited the following potential advantages: (1) better biocontainment of the chimeric virus; (2) better conformation of virions; (3) increased payload on the CVPs; (4) higher cost-effectiveness in the production process; and (5) faster development of new vaccine candidates. The reason for better containment of the chimeric virus is that the coding region for TGBps, involved in virus movement, on the chimeric BaMV genome was replaced by that of the TEV protease, rendering the chimeric BaMV unable to move out of the inoculated regions in plants. Therefore, the biosafety concern for the chimeric virus could be reduced. For better virion conformation, the reason is that the CP of the chimeric virus is essentially the same as that of the original BNd35, except for 5 extra glycine residues at the N-terminus following the cleavage by TEV protease. As shown in **Figure 5**, the morphology of B5G and BJLPET5G virions was indistinguishable from that of BNd35. Similarly, the payload of the current system is higher (meaning that B5G could be applied for the presentation of larger epitopes), since the epitopes were ligated after the formation of virions. Thus, the interference of large sizes of epitopes on CVPs assembly was eliminated. Our new system is more cost-effective, since the TEV protease is directly produced by the chimeric virus in plants, and the processing of CP and auto-assembly of the CVPs were completed within the plants. In addition, the unreacted rEDIII-CaM and SrtA proteins could be recovered from the Ni^2+^-NTA column and reused in another ligation reaction, reducing the cost for the production of SrtA-ready B5G and target epitopes. In the current binary system, the production of B5G CVPs in plants is separated from that of the target antigens in bacteria. The B5G CVPs could be mass-produced in the greenhouse or fields following the regulation of Current Good Manufacturing Practice (cGMP), tested for quality control, and stored or applied immediately by SrtA-mediated ligation with desired antigens for the development of other vaccine candidates. In contrast, in the previous CP-epitope fusion approaches, such as (14), a new chimeric virus construct has to be generated for every new epitope, some of which may severely interfere with the infectivity or assembly of the chimeric virus. Therefore, the current binary system requires less time and efforts in the development cycles of new vaccine candidates. Furthermore, the results of immunization assays revealed that BJLPET5G stimulated the production of neutralizing antibodies with higher titers against JEV infection.

### Possible Means for Further Optimization of the SrtA-Mediated Ligation

It has been reported that the coupling density of M2e peptide onto the PapMV-N nanoparticles (made of PapMV CP with N-terminal acceptor site) could reach up to 83% by increasing the incubation time and the concentration of M2e peptide (22). However, in our system, the efficiency of SrtA-mediated ligation was only around 20% to 40% (**Figures 3C, F** and **4D**). One of the major reasons might be the reversibility of SrtA-mediated ligation, in which the ligation of donor and acceptor restored the original SrtA recognition sequence LPETG-GS, which could be re-cleaved by SrtA. The evidence for our reasoning is that the longer incubation time for SrtA reaction resulted in less yield of antigen-coupled B5G CPs (**Figures 3A, D**), suggesting that the ligated rEDIII proteins were re-cleaved by SrtA. Several methods have been developed to minimize the reversibility of the SrtA-mediated ligation [as comprehensively reviewed by Antos et al., (41)]. The first type of approaches is to increase the supply of either the donor or acceptor. The second is the separation of desired ligation product from the by-products by selective removal of the cleaved amino-glycine peptide fragment by dialysis, centrifugal filtration, or affinity immobilization (42–47). As for our system, the B5G CVPs provide a highly ordered arrangement of a large amount of CPs as receptors [estimated to be about 1150 copies per virion, based on structure of BaMV (39)], which should have driven the reaction towards the formation of BJLPET5G. However, the coupling efficiency was not as high as expected, suggesting that the increase of acceptor supply was not sufficient to increase the coupling efficiency. In addition, the ratio among B5G, rEDIII, and SrtA also plays a crucial role in the coupling reaction (**Figure 3**). Thus, the increase of the amount of one reactant might have resulted in the decrease in coupling efficiency (**Supplementary Figures 7A, B**). Another type of approach is the de-activation of the ligation product or the by-product through modification of the donor and acceptor sequences to form unreactive β-hairpin around the SrtA recognition site or to release of unreactive by-products following the desired ligation (48–54). These de-activation strategies could be applied in further studies to enhance the coupling efficiency of the current system. On the other hand, it has been shown that particles of certain plant viruses may serve as the adjuvant in vaccination (55). *Potato virus X* (PVX), the type species of the genus *Potexvirus*, is one of the viruses with adjuvant potential (10). BaMV is also a member of the genus *Potexvirus*, with similar virion morphology, suggesting that even if some BaMV virions were not labeled with rEDIII, these virions may serve as adjuvants in the vaccine preparations.

### Unique Features of the Current Binary System

In contrast to the previous designs, our current system exhibits several unique features. Firstly, the BaMV CVP scaffolds are produced in plants, as opposed to those produced in bacteria or animal cells (16–18, 21, 22, 56, 57). The plant production system reduced the risk of contamination from prokaryotic or animal sources, and allows for easy scale-up. The requirement for the inoculation process could be eliminated by using transgenic approaches. Previously, we have developed transgenic *N. benthamiana* for the production of BaMV-based vaccine candidates (40). With simple modifications, transgenic *N. benthamiana* expressing B5G CVPs could be generated, which would also compensate for the deletion of TGBps (viral movement proteins) in the B5G construct and ensure the production of B5G virions in each cell of the transgenic plants. Secondly, our design facilitated auto-processing of CP and self-assembly CVP scaffold. In comparison, the scheme for PapMV-based platform recently published (22) requires pH and temperature shift with buffer exchange and an incubation time of 16-24 hrs for intein removal following the harvesting of PapMV CP from the bacterial cells. Thirdly, our system provides a vector for the expression of target antigens with improved solubility by fusing with the 6×His-CaM-6×His tag, which could be removed upon ligation with the B5G CP by SrtA. In our previous study (14), the solubility of rEDIII expressed in bacterial cells was only moderate, leading to significant loss in the purification process and the requirement for re-solubilization. Our current design enhanced the solubility of target proteins, and facilitated the separation of target protein-labeled B5G CVPs from the cleaved 6×His-CaM-6×His tag and the unreacted target proteins. Lastly, our system allowed for highly efficient SrtA-mediated ligation reaction, which could be completed in 1 min (**Figures 3C, F**), as opposed to the previous systems which require hours of reaction time (16–18, 21, 22, 56, 57). All these features contributed to the applicability of the current system.

The platform presented in current study would produce CVPs with sizes in the range of 488-504 nm, which possibly affect the efficiency of lymph node drainage as they required the assistance of dendritic cells (DCs) to enter the lymphatic vessels (5). However, in a previous study, PVX particles, with similar sizes as BaMV virions, were used as the scaffold to be conjugated with the antigen of interest, and the results of immunization assays showed promising levels of both B-cell and T-cell stimulatory effect (58). In addition, we have previously developed BaMV-based epitope presentation platform to produce vaccine candidates against FMDV and IBDV, and demonstrated the efficient stimulation of both humoral and cellular immune responses against target antigens in swine and chickens (11, 13). The vaccinated chickens showed similar protective effect as commercially available D78 vaccine in the IBDV challenges (13). It is also worth noting that the larger particles are advantageous in presenting more antigens, and with the assistance of proper adjuvants could form a local depot at the injection site, resulting in prolonged stimulation of immune system to build up the similar protective effect (5).

## Conclusions

An alternative approach for the production of BaMV-based CVPs was provided, facilitating the presentation of antigens (rEDIII and VP1 peptide) through SrtA-mediated coupling. Our approach consists of two subsystems: one for the production of SrtA-ready B5G CVPs in *N. benthamiana*, the other for the generation of target antigens and SrtA in *E. coli*. Upon mixing the B5G CVPs with the antigen proteins and SrtA, antigen-presenting CVPs (BJLPET5G) could be generated in 1 min and readily purified by removing the unreacted antigen proteins and SrtA through Ni^2+^-NTA column. Injection of mice with the BJLPET5G CVPs resulted in the stimulation of immune responses against JEV, demonstrating the prospective applicability of our approach. This study thus provides an efficient system for the development of vaccine candidates, adding to the arsenal against the ever-emerging pandemics.

## Data Availability Statement

The datasets presented in this study can be found in online repositories. The names of the repository/repositories and accession number(s) can be found in the article/**Supplementary Material**.

## Ethics Statement

This animal protocol was approved by the Academia Sinica Institutional Animal Care and Use Committee (Protocol no. 17-11-1123) and was performed in accordance with the guidelines.

## Author Contributions

M-HY performed the SrtA-ready BaMV construction, the coupling experiments on the B5G BaMV platform, batch production of coupling CVPs, and was involved with immunoelectron microscopy and animal experiments. M-HY also drafted the manuscript. C-CH, C-HW, N-SL, Y-HH, and Y-LL helped draft the article structure of the manuscript. J-JL helped perform mouse experiments. H-YK provided technical assistance for animal experiments and useful advice and discussion. M-HH helped perform immunoelectron microscopy experiments. Y-LL and N-SL guided and provided advice and discussion of the animal experiences and the immunoelectron microscopy. Y-HH supervised the entire study, organized the participation of each lab participant. All authors contributed to the article and approved the submitted version.

## Funding

This work was supported by grants from the Ministry of Science and Technology, Taiwan (MOST-109-2313-B-005-050 to Y-HH; MOST-108-2320-B-001-030-MY3 to Y-LL), and by the Advanced Plant Biotechnology Center from The Featured Areas Research Center Program within the framework of the Higher Education Sprout Project by the Ministry of Education (MOE), Taiwan.

## Conflict of Interest

The authors declare that the research was conducted in the absence of any commercial or financial relationships that could be construed as a potential conflict of interest.

## Publisher’s Note

All claims expressed in this article are solely those of the authors and do not necessarily represent those of their affiliated organizations, or those of the publisher, the editors and the reviewers. Any product that may be evaluated in this article, or claim that may be made by its manufacturer, is not guaranteed or endorsed by the publisher.

## Supplementary Material

Supplementary Figure 1 Analysis of the expression of rEDIII in *E. coli*.

Supplementary Figure 2 Analysis of SrtA-mediated coupling reaction.

Supplementary Figure 3 Comparison of rEDIII decoration on various BaMV CVPs through immunoelectron microscopy.

Supplementary Figure 4 Comparison of sera titers. Five 3-week-old female BALB/c mice per group were immunized with B5G, rEDIII, or BJLPET5G.

Supplementary Figure 5 Comparison of titers in serially diluted sera at each time point.

Supplementary Figure 6 Reporter virus neutralization test (RVNT) using eGFP-modified Japanese encephalitis virus (JEV-eGFP) in BHK-21 cells.

Supplementary Figure 7 Analysis of the coupling efficiency by adjusting the amount of B5G CP.

Supplementary Figure 8 Construction of VP1 peptide with TrxA fusion tag for *E. coli* expression.

Supplementary Figure 9 Application of the B5G CVP system in presenting VP1 peptide.

Supplementary Table 1 Sequences of Amplified Primers

## Abbreviations

AlMV, alfalfa mosaic virus; BaMV, bamboo mosaic virus; B5G CP, BaMV coat protein with 5 protruding G residues; B5G CVP, chimeric virus particle made of B5G coat protein; BJLPET5G, rEDIII-presenting BaMV chimeric virus particles; BVP1LPET5G, VP1 peptide-presenting BaMV chimeric virus particles; CaM, calmodulin; CBS, Coomassie Blue Staining; cGMP, Current Good Manufacturing Practice; CP, coat protein; CVPs, chimeric virus particles; DAPI, 4’, 6-diamidino-2-phenylindole; DCs, dendritic cells; DPI, days post-inoculation; EGTA, ethylene glycol tetraacetic acid; ELISA, enzyme-linked immunosorbent assay; FMDV, foot-and-mouth disease virus; G, glycine; GraphPad, GraphPad Prism version 6.0; HRP, horseradish peroxidase; IBDV, infectious bursal disease virus; JEV, Japanese encephalitis virus; JEV-eGFP, JEV (RP-9) expressed enhanced green fluorescent protein; *N. benthamiana*, *Nicotiana benthamiana*; NBT/BCIP, Nitro-blue tetrazolium/5-bromo-4-chloro-3’-indolyphosphate; PapMV, papaya mosaic virus; PCR, Polymerase chain reaction; PEG, polyethylene glycol; PVX, potato virus X; rEDIII, recombinant envelope protein domain III; rEDIII-mCP, rEDIII-LPET-5G-mCP; RVNT, reporter virus neutralization test; SDS-PAGE, polyacrylamide gel containing 1% sodium dodecyl sulfate; SrtA, sortase A; TEV, tobacco etch virus; TGBps, triple-gene-block proteins; TM, TEV protease cleavage motif (ENLYFQG); VLPs, virus-like particles; 4G, tetra-glycine; 6×His, 6×His-tagged; 7×His-TEV protease, histidine-tagged TEV protease.
