# Supplementary material for: Convenient Auto-Processing Vector Based on Bamboo Mosaic Virus for Presentation of Antigens Through Enzymatic Coupling
Source: Front Immunol. 2021 Oct 14;12:739837. doi: 10.3389/fimmu.2021.739837 (PMC8551676; doi:10.3389/fimmu.2021.739837)
Supplement: Supplementary Figure 1 — Analysis of the expression of rEDIII in E. coli. [file DataSheet_1.pdf]

Supplementary Material

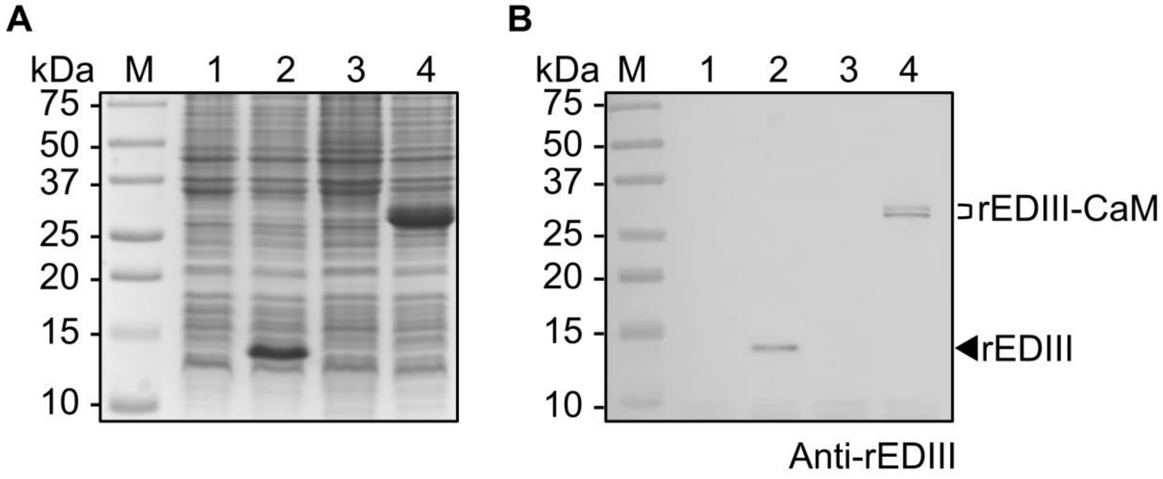

Supplementary Figure 1. Analysis of the expression of rEDIII in *E. coli*. (A) SDS-PAGE analysis of bacterially expressed rEDIII and rEDIII fused with calmodulin (CaM) (rEDIII-CaM) proteins. *E. coli* BL21 cells were transformed with pET21d-rEDIII, pET29a-rEDIII-CaM, and incubated at 37 °C until optical density at 600 nm (OD_{600}) reached 0.8, followed by induction of protein expression with IPTG (0.4 mM) at 28°C for 4 h. Protein samples were analyzed by 15% SDS-PAGE and visualized by CBS. The proteins were then electro-blotted to PVDF membranes, and probed with antiserum specific to JEV rEDIII (B). Lanes 1, and 2, rEDIII before and after IPTG induction, respectively; lanes 3 and 4, rEDIII-CaM before and after IPTG induction, respectively; lane M, molecular weight markers. The positions of rEDIII (≈12 kDa) and rEDIII-CaM (≈32 kDa) are indicated by the arrowhead and bracket on the right, respectively.

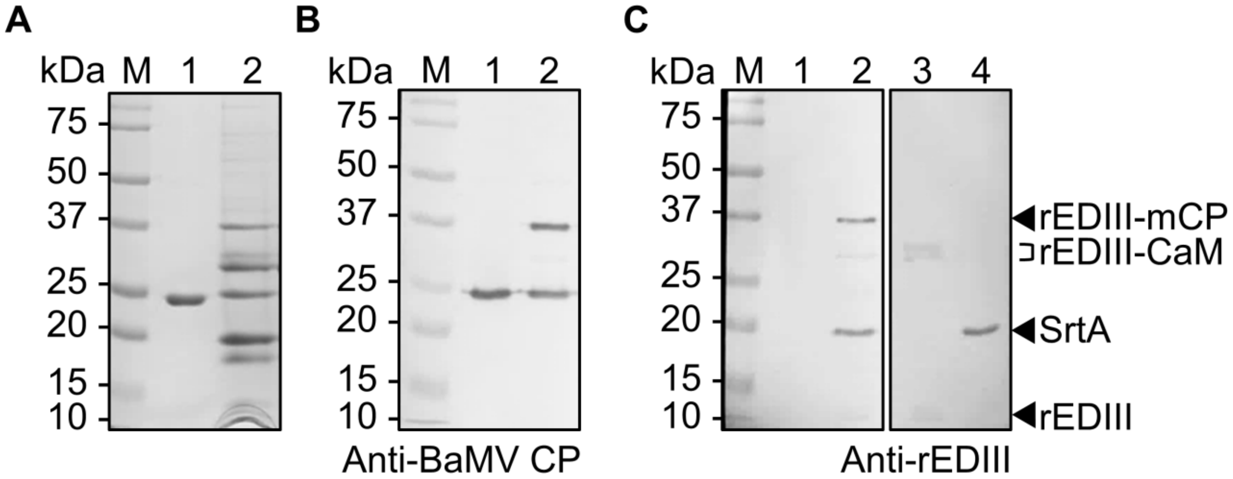

Supplementary Figure 2. Analysis of SrtA-mediated coupling reaction. (A) SDS-PAGE analysis SrtA reaction products. Protein samples were analyzed by 12% SDS-PAGE and visualized by CBS. The proteins were then electro-blotted to PVDF membranes, and probed with antiserum specific to BaMV coat protein (B), or JEV rEDIII (C). Lanes 1; B5G alone; 2, unpurified SrtA reaction products; lanes 3 and 4, rEDIII-CaM alone and SrtA alone, respectively; lane M, molecular weight markers. The positions of rEDIII-mCP (≈35 kDa), rEDIII-CaM (≈32 kDa), SrtA (≈18 kDa), and rEDIII (≈12 kDa) are indicated by the arrowheads and bracket on the right. The polyclonal antibody against rEDIII may also react with SrtA protein, resulting in a signal in panel (C), lane 4.

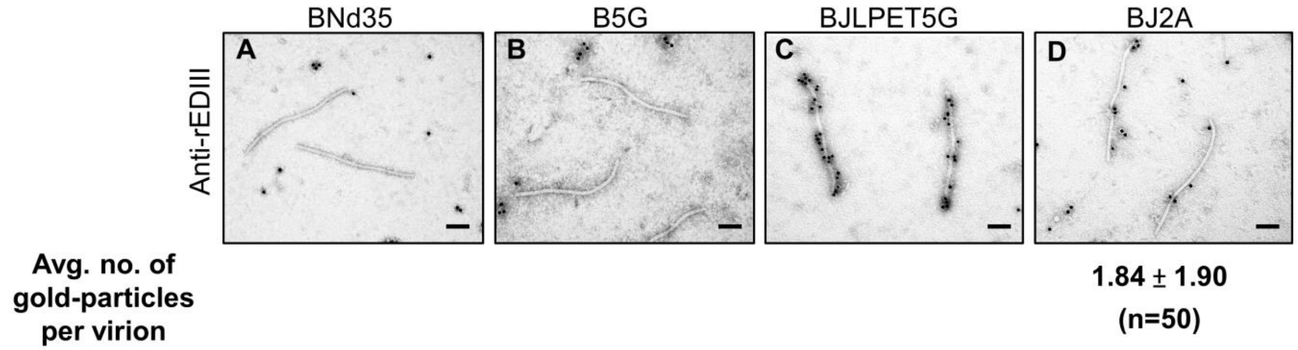

Supplementary Figure 3. Comparison of rEDIII decoration on various BaMV CVPs through immunoelectron microscopy. Purified CVPs of BNd35 (A), B5G (B), BJLPET5G (C), and BJ2A (D) were examined by transmission electron microscopy using antisera specific to JEV rEDIII followed by labeling with the gold-conjugated goat anti-rabbit IgG secondary antibodies. Average numbers of gold particles on BJ2A CVP surface (n = 50) treated with antisera-specific rEDIII are indicated below panel (D). Scale bars, 100 nm.

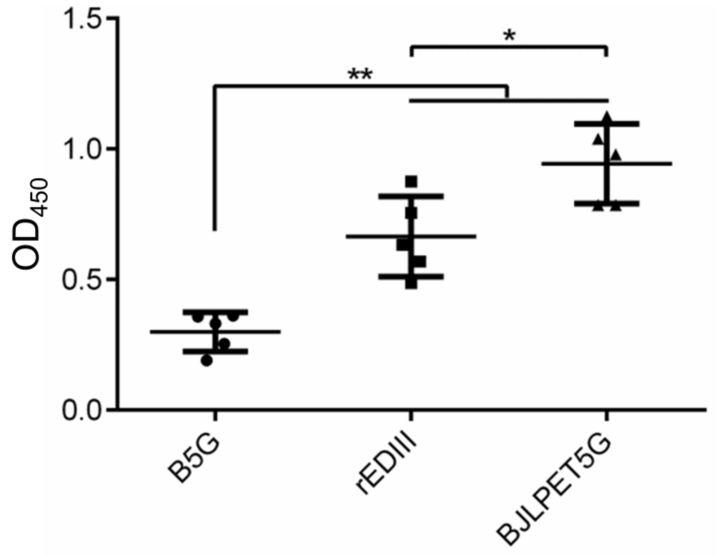

Supplementary Figure 4. Comparison of sera titers. Five 3-week-old female BALB/c mice per group were immunized with B5G, rEDIII, or BJLPET5G. The antibody titers of sera collected from the third bleeding were analyzed. The scatter chart illustrates the distribution of OD_{450} values in the ELISA with serum sample from individual mouse. B5G and rEDIII were used as the negative and positive control, respectively. The standard deviations are indicated by the error bars. Statistical significance was analyzed using two-tailed Student's *t* tests; *, $p < 0.05$; **, $p < 0.01$.

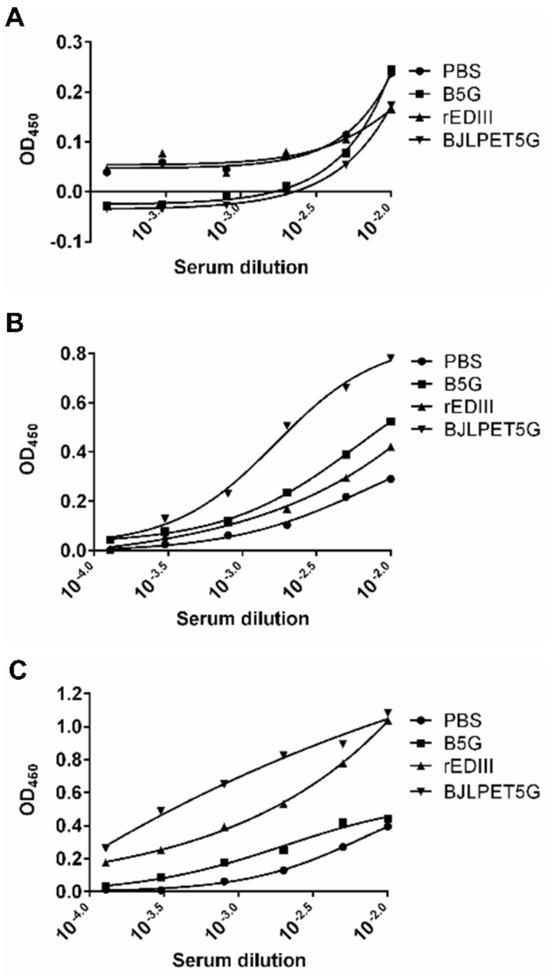

Supplementary Figure 5. Comparison of titers in serially diluted sera at each time point. The serum was collected at different stages as indicated in Fig. 6A, including pre-immune (A), priming (B), and boosting (C). The sera from mice of the same group were pooled, serially diluted, incubated with JEV-infected (RP-9) C6/36 cells coated on plates, and analyzed for antibody titers in an enzyme-linked immunosorbent assay (ELISA). The charts represent the values of optical density at 450 nm (OD_{450}) obtained in the ELISA. The standard curves are determined by nonlinear, dose-response regression analysis with GraphPad Prism version 6.0 (GraphPad, San Diego, CA, USA).

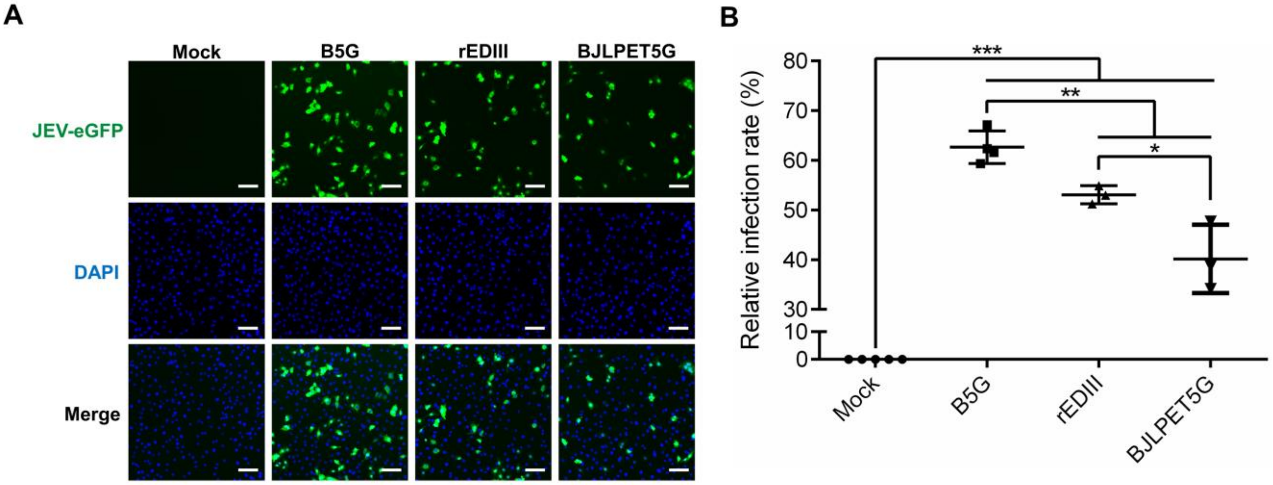

Supplementary Figure 6. Reporter virus neutralization test (RVNT) using eGFP-modified Japanese encephalitis virus (JEV-eGFP) in BHK-21 cells. (A) JEV-eGFP were reacted with individual serum sample (250-fold dilution) from the immunized groups of mice, and incubated with BHK-21 cells for 2 h, followed by examination under fluorescent microscope. The infected cells were indicated with eGFP fluorescence (green), and the cell nuclei were stained with DAPI (blue). The activity of neutralizing antibodies was examined in triplicate. Scale bars, 100 µm. The relative infection rate was shown in the scatter chart (B), which illustrates the mean values of experiments performed in triplicate from nine fields per well observed with serum from individual mouse. B5G and rEDIII were used as the negative and positive control, respectively. The standard deviations are indicated by the error bars. Statistical significance was analyzed using two-tailed Student's *t* tests; *, $p < 0.05$; **, $p < 0.01$; ***, $p < 0.001$.

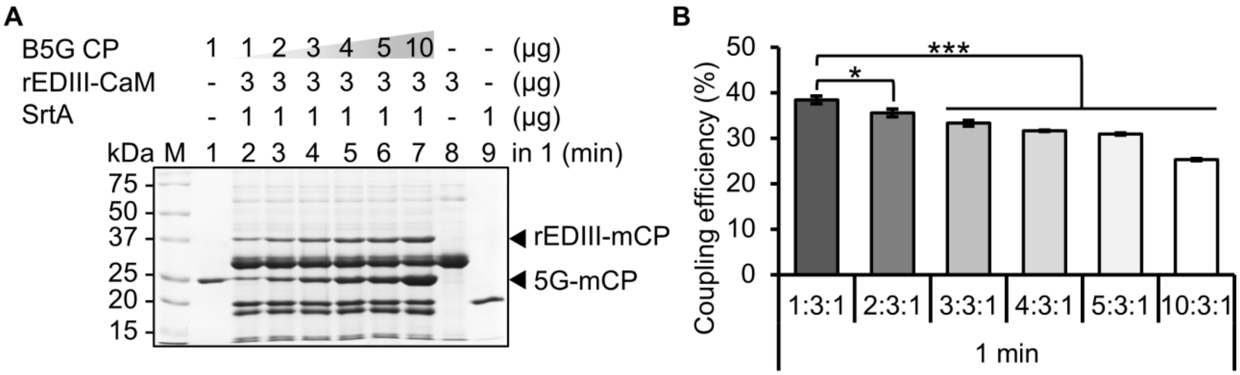

Supplementary Figure 7. Analysis of the coupling efficiency by adjusting the amount of B5G CP. (A) Effect of different ratios of B5G CP. The amount of CP was increased by 2 to 10 folds, as indicated on top of each lane, relative to that of SrtA, with a reaction time of 1 min. The components in each reaction are shown on top of each lane. Protein samples were analyzed by 12% SDS-PAGE, and the protein bands were visualized by CBS. The expected sizes of different forms of proteins were indicated by the arrowheads on the right. The relative coupling efficiencies under different reaction conditions were shown in the bar chart (B). The relative intensities of conjugated rEDIII-CP and unconjugated CP were individually quantified using the software ImageJ and presented using Microsoft Excel for each reaction condition. Statistical significance was analyzed using two-tailed Student's *t* tests; *, $p < 0.05$; **, $p < 0.01$; ***, $p < 0.001$. Error bars represent the standard deviation of three independent experiments.

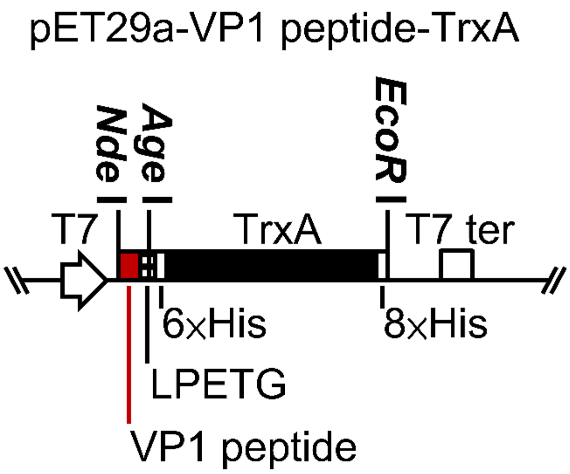

Supplementary Figure 8. Construction of VP1 peptide with TrxA fusion tag for *E. coli* expression. Schematic of the construct, pET29a-VP1 peptide-TrxA, for the expression of VP1 peptide fused with thioredoxin (TrxA) (VP1 peptide-TrxA). The blank arrow and box represent the T7 promoter (T7) and T7 terminator (T7 ter), respectively.

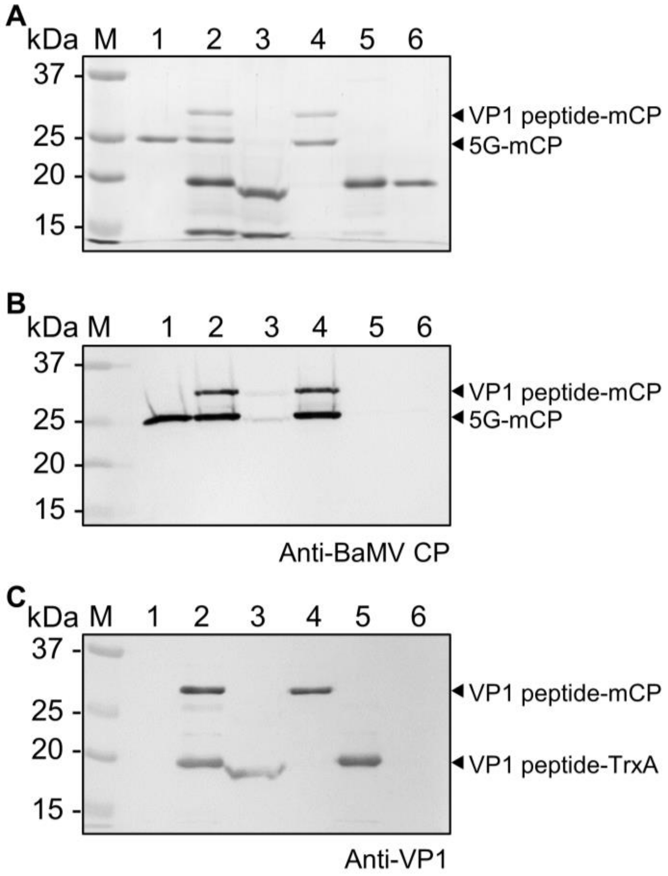

Supplementary Figure 9. Application of the B5G CVP system in presenting VP1 peptide. The VP1 peptide was used as the target to be displayed on B5G CVPs as described, and the resulting CVPs were analyzed. (A-C) SDS-PAGE and western blot analysis of BVP1LPET5G CVPs purified through PEG 6000 precipitation. The proteins were separated by 12% SDS-PAGE, visualized by CBS (A), or electro-blotted to PVDF membranes and probed with antiserum specific to BaMV coat protein (B) or VP1 protein (C). Lanes 1, B5G alone; lanes 2, the supernatant, obtained through a low-speed centrifuge after EGTA treatment; lanes 3 and 4, the supernatant and pellet collected after PEG precipitation. Purified VP1 peptide-TrxA (lane 5) and SrtA (lane 6) proteins were used as size markers. The positions of each protein are indicated by the solid arrowheads on the right.

Supplementary Table 1. Sequences of Amplified Primers

Primers (Restriction enzyme)	Sequences (5'-3')
TM-4G-F (*Age*I)	GGGACCGGTATGgagaacctgtatttccagggtgg
TM-4G-R (*Not*I)	GGGTGCGGCCGCaccaccaccaccaccctgg
7×His-TEV protease-F (*Dra*III) (TEVp-F)	GGGCACGCGGTGATGcatcaccatcaccatcaccatggtgaaagc
TEV protease-stop-R (*Dra*III)	CCCCACATGGTGTCAattcatgagttgagtcgcttccttaactgg
6×His-CaM-F (*Nde*I)	GGGCATATGcaccaccaccaccaccacgcagagcagctaacggaggag
CaM-GSS-6×His-R (*Eco*RI)	GGGGAATTCTTAgtggtggtggtggtggtgagaagaacccttggcaagcatcatg
rEDIII-F (*Nde*I)	GGGCATATGgacaaactggccctgaaaggc
rEDIII-GSS-LPETG-GS-R (*Nde*I)	GGGCATATGagaaccaccagtctctggcagagaagaacccgtgcttccagctttg
TGSS-TrxA-6×His-F (*Age*I)	GGGACCGGTggttctcaccaccaccaccaccacagcgataaaattattc
TrxA-8×His-R (*Eco*RI)	CTCGAATTCtcaatgatgatgatgatggtggtgatggccagaaccagaaccggccaggttag
VP1-F (*Nde*I)	GGGCATATGaccgtctacaacggaagcag
VP1-GSS-LPETG-R (*Age*I)	ACCACCGGTctctggcagagaagaaccgttgaaag
4102-F	ccactaccaaacaatcag
5703-R	atccactgcttagtgtttgg

The restriction enzyme site and corresponding gene fragment were underlined or shown in lower case, respectively. TEV motif, *Tobacco etch virus* cleavage sites; His, Histidine; CaM, Calmodulin; rEDIII, Recombinant envelope domain III; GSS, peptide linker; LPETG-GS, SrtA recognition site; stop, Stop codons
